# Finite-Element Analysis of Bone Stresses on Primary Impact in a Large-Animal Model: The Distal End of the Equine Third Metacarpal

**DOI:** 10.1371/journal.pone.0159541

**Published:** 2016-07-26

**Authors:** Cristin A. McCarty, Jeffrey J. Thomason, Karen D. Gordon, Timothy A. Burkhart, Jaques S. Milner, David W. Holdsworth

**Affiliations:** 1 Department of Biomedical Science, Ontario Veterinary College, University of Guelph, Guelph, Ontario, Canada; 2 Department of Biomedical Engineering, School of Engineering, University of Guelph, Guelph, Ontario, Canada; 3 Department of Mechanical and Materials Engineering, School of Engineering, Western University, London, Ontario, Canada; 4 Robarts Research Institute, Schulich School of Medicine and Dentistry, Western University, London, Ontario, Canada; 5 Department of Surgery, Schulich School of Medicine and Dentistry, Western University, London, Ontario, Canada; Université de Lyon—Université Jean Monnet, FRANCE

## Abstract

**Objective:**

To assess whether the transient stresses of foot impact with the ground are similar to those found during midstance loading and if the location of high stress correlate with the sites most commonly associated with mechanically induced osteoarthritis (OA). We compared impact stresses in subchondral bone between two subject-specific, three-dimensional, finite-element models of the equine metacarpophalangeal (MCP) joint—one with advanced OA and one healthy, and with similar published data on the stresses that occur at midstance.

**Methods:**

Two right MCP joints (third metacarpal and proximal phalanx) were scanned using micro-computed tomography (μCT). Images were segmented, and meshed using modified 10-node quadratic tetrahedral elements. Bone material properties were assigned based on the bone density. An impact velocity of 3.55 m/s was applied to each model and contact pressures and stress distribution were calculated for each. In a separate iteration, the third metacarpal was loaded statically. A sampling grid of 160 equidistant points was superimposed over selected slices, and average peak stresses were calculated for 6 anatomical regions. Within-region maximal peak and average von Mises stresses were compared between healthy and OA bones in both midstance and impact loading.

**Results:**

Average impact stresses across all regions, in both locations (palmar and dorsal) were greater in the OA model. Highest impact stresses were located in the dorsal medial condyle in the healthy (12.8 MPa) and OA (14.1MPa) models, and were lowest in the palmar medial and lateral parasagittal grooves in the healthy (5.94 MPa) and OA (7.07 MPa) models. The healthy static model had higher peak (up to 49.7% greater) and average (up to 38.6% greater) stresses in both locations and across all regions compared to the OA static model.

**Conclusions:**

Under simulated footfall a trot, loading on the dorsal aspect of the third metacarpal at impact created stresses similar to those found during midstance. The high accelerations that occur under impact loading are likely responsible for creating the high stresses, as opposed to midstance loading where the high stresses are the result of high mass loading. Although the stress magnitudes were found to be similar among the two loading conditions, the location of the high stress loading occurred in sites that are not typically associated with osteoarthritic changes.

## Introduction

Mechanical loading of joints is known to be a factor in the development of osteoarthritis (OA). In horses, forces on each limb at the faster gaits rise and fall approximately sinusoidally from first to last contact of the foot with the ground, peaking at the halfway point (midstance). This represents a high amplitude, low-frequency (<10Hz) loading regime, and previous studies have focussed on the high midstance stresses as primary candidates in the etiology of OA. As the hoof makes first contact with the ground, however, there is a 3-10ms period—called primary (1°) impact—during which transient loading occurs, of lower amplitude but higher frequency (≥100Hz).

This study presents a preliminary assessment of the transient impact stresses, to assess how their magnitudes and distribution in a joint condyle compare between a healthy and osteoarthritic bone and with previously published data for midstance stresses at the same location. The aim is simply to ask whether the transient stress magnitudes and distribution on 1° impact warrant further investigation in the context of the mechanical etiology of OA.

**Fig 1:**
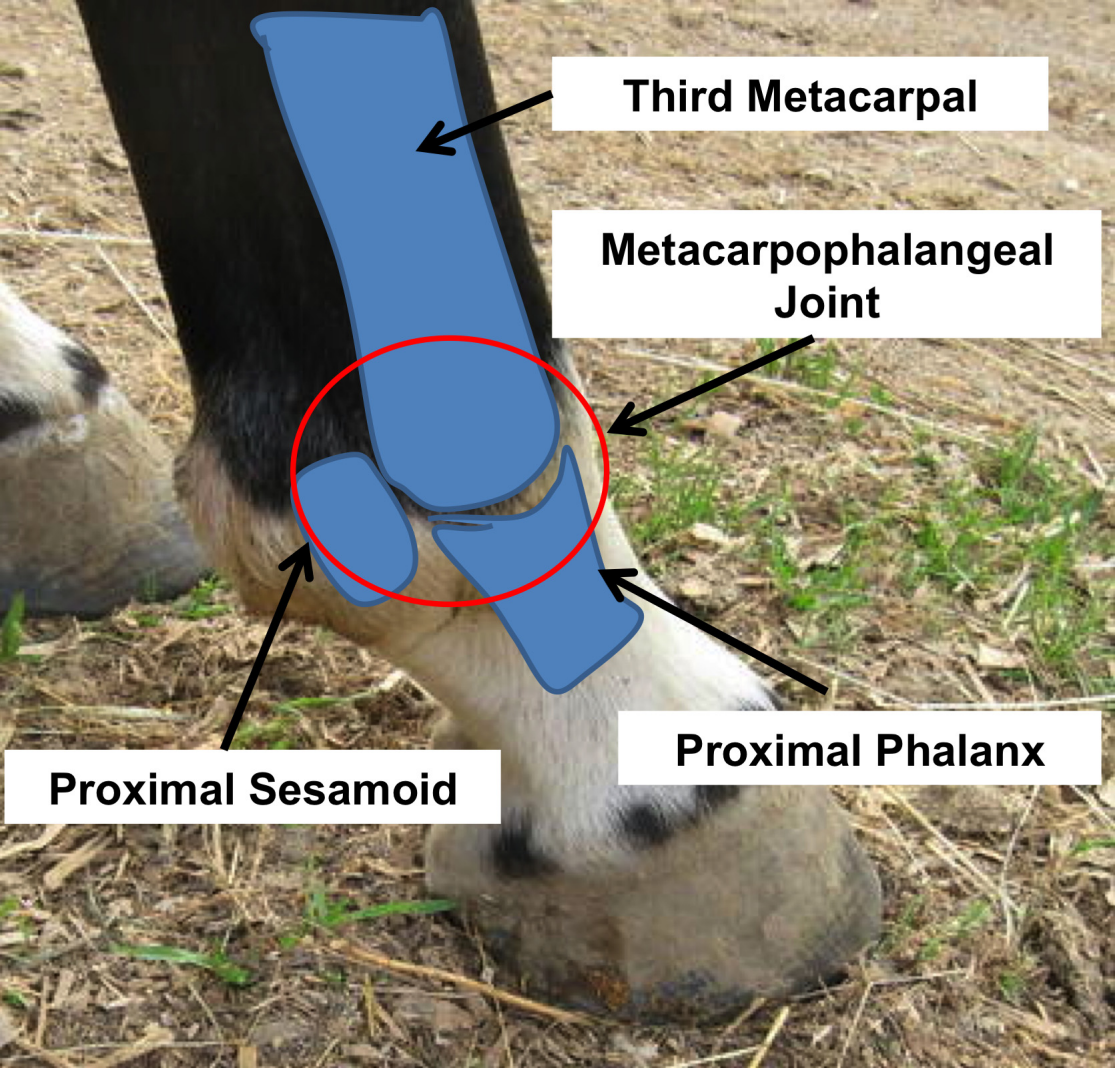
Equine Metacarpophalangeal Joint. Anatomy of the equine MCP joint.

**Fig 2:**
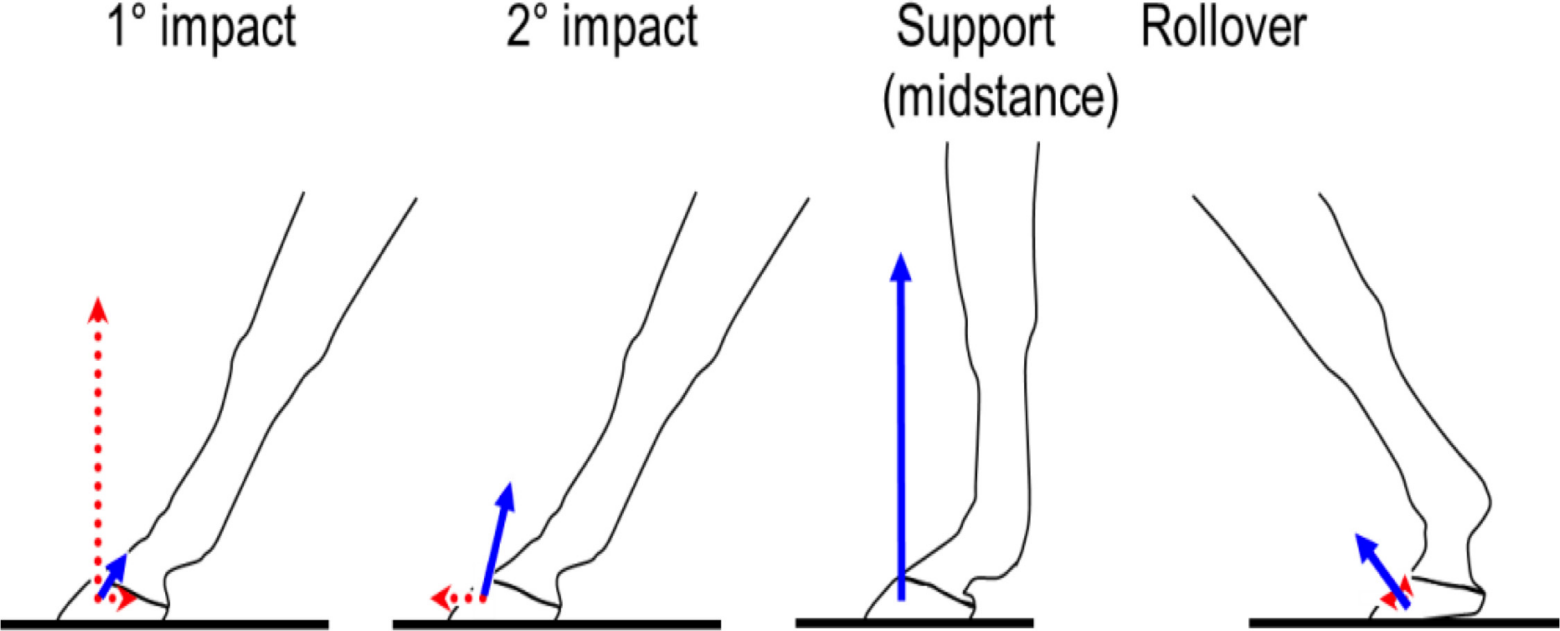
Phases of the stance and associated loading conditions at each phase. Red arrows indicate vertical and horizontal acceleration and blue arrows indicate the ground reaction forces associated at each phase of the stance. The length of the arrow represents the magnitude of the acceleration and/or force at a given phase. Figure has been reproduced from Thomason and Peterson 2008 with permission.

**Fig 3:**
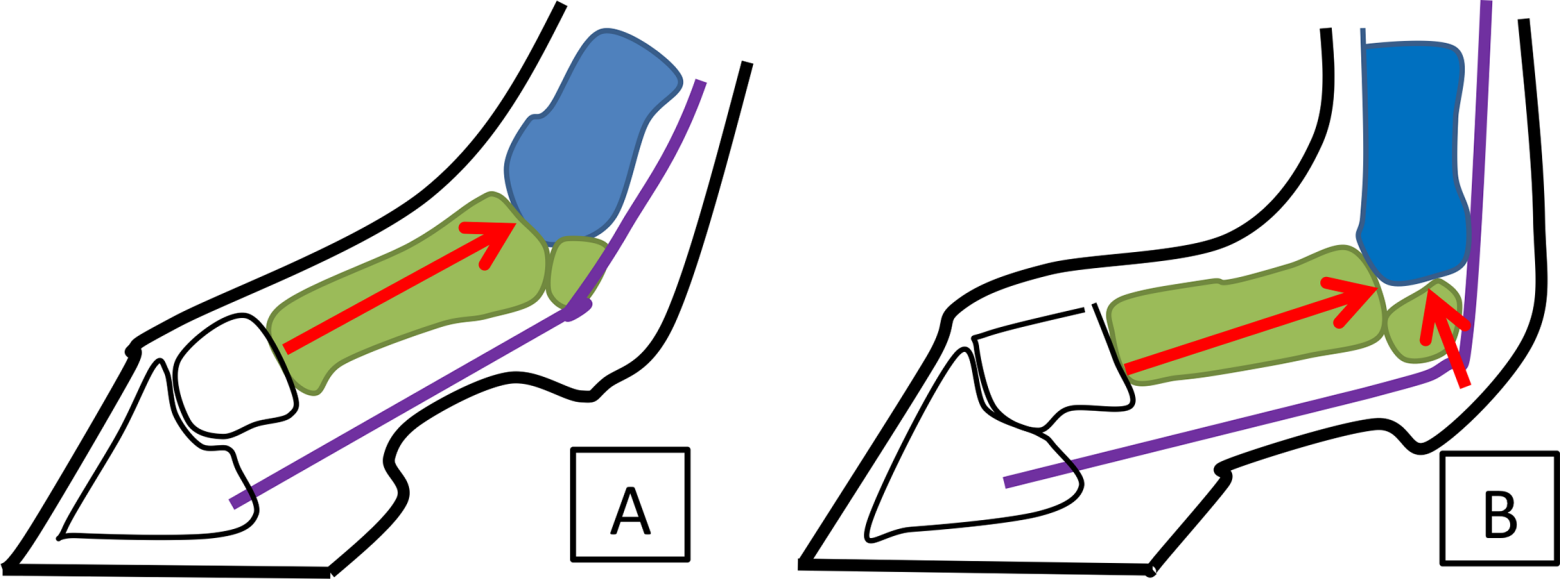
Loading on the MCP joint at Impact and Midstance phases of the stance. Red arrows indicate loading on the distal end of MC3 from P1 under 1° impact [A] and the loading from both P1 and the PS under midstance [B]. Note the increased tension in the deep digital flexor tendon that occurs under midstance loading as indicated by the purple line.

The metacarpophalangeal (MCP) joint of horses (Fig 1) is a suitable model for asking this question based on the following: 1) the condyle of the equine third metacarpal (MC3) is a common site of injury and OA, 2) 1° impact and midstance loading are clearly separated temporally during each stance (Fig 2), and 3) the high stresses in MC3 that occur during midstance are located in sites common to injury, including OA and are generally thought to play a key role in the joint changes associated with OA [1,2]. If impact stresses are of similar magnitude at the sites where injury occurs, they may also be implicated in the etiology of OA. Some injuries to the MCP and MC3 are thought to be due to overuse and repetitive loading, eventually leading to degeneration of the joint and chronic lameness. Osteoarthritis in the MCP joint is common among Standardbred and Thoroughbred racehorses [3,4] and is associated with a change in the micro-architecture of the subchondral bone and overall joint geometry [2,5,6].

The underlying bone structure within the MCP joint is representative of the mechanical loading history (dependent on magnitude, rate and repetitiveness) sustained during high-speed racing and training [7,8]. The joint experiences a high range of motion during the stance, from being approximately straight at 1° impact to as much as 90° of hyperextension at midstance. Loading is distributed over a relatively small surface area (in comparison to the horse’s body size) and involves multiple loading sites as the joint is progressively hyperextended while under load, before unloading to enter the swing phase.

Contact stresses at midstance (Fig 2) (i.e., at full joint extension) represent the peak of high amplitude, low frequency loading on the joint. They have been shown to be associated with site-specific changes within the distal end of MC3 [8,9]. Previous finite element modeling of the mechanics of the MCP joint have shown that the condyles of MC3 undergo loading in both the dorsal and palmar aspect from the first phalanx (P1) and proximal sesamoid bones (PS) at midstance (Fig 3), creating a combination of high compressive and shear loading on MC3 [1,2].

During 1° impact, the combination of high accelerations and low masses (the distal portion of the limb) are responsible for the force generation at this stage of the stance. Force magnitudes are low during 1° impact (~2–10% of the peak at midstance), but it is known that repetitive impact can be involved in the etiology of OA [10,11,12]. Accelerations of high magnitude and high frequency have also been shown to elicit bone changes and contribute to damage within a joint [11].

Repetitive loading under high-speed locomotion occurs 100–150 times per minute while horses are racing and training, and imposes large stresses on the MCP joint. Over time, stresses such as this are known to cause microcracks in the subchondral bone (SCB) and calcified cartilage, and can initiate bone modeling [10]. Modeling typically increases the bone density in an effort to resist overloading and subsequent failure. Based on the mechanical properties of bone, more dense bone is generally stiffer than bone that is less dense [13] when loaded. Some horses with OA develop a persistent resorptive response where bone formation and resorption are uncoupled, resulting in remodeling that is sustained for months and can lead to irreversible and permanent loss of bone architecture [14]. This dysregulation of bone metabolism leads to focal radiolucent areas that can undermine and penetrate the SCB plate resulting in a loss of SCB support and invasion into the calcified cartilage, weakening the internal structure of the bone through crack propagation by increasing porosity in the area [15].

Characterization of stresses associated with the loading conditions at all stages of the stance (Fig 2) is essential in understanding the biomechanical loading that may be associated with the changes in bone tissue. Given the complexities that are associated with measuring the joint forces and contact areas *in vivo*, computational models, specifically finite element models, are practical alternatives. Finite element modeling (FEM) provides a method for predicting the stresses and strains in structures that have complex geometries, specific material properties and that are subjected to complex loading patterns. Finite element modeling has been used within equine research to examine the stress distribution at midstance in the hoof [16,17] proximal phalanx [1,18], third metacarpal [1,2,19] and entire distal forelimb [20]. While these studies have provided valuable data including the stress distribution within the MCP joint under quasi-static loading associated with midstance, there is currently no equine FEM that examines the stresses under 1° impact loading. Therefore, the purpose of this study was to create two subject-specific (in terms of bone geometry, structure and corresponding material properties), three-dimensional, finite-element models of the equine MCP joint (one with advanced OA and one healthy) and compare the stresses between the two models and examine and compare stresses that occur under 1° impact to those that occur during midstance loading. Additionally, we wanted to examine the how the stress distribution under impact and midstance loading changed within the changes in subchondral bone density and bone architecture as observed in horses with OA. We expect that the location of the high stresses among the healthy and OA model will differ between the 1° impact and midstance loading. Additionally, we expect that bone density and bone architecture changes present in the OA model will have an effect when compared to the stresses of the healthy FEM.

## Materials and Methods

An ethical committee review was not required as this study used cadaver limbs that were severed post-mortem from animals that has either died of natural causes or had been euthanized for some other reason unrelated to this study. All animals were registered within the Ontario Death Registry conducted by the Racing Commission of Ontario and were subject to routine post-mortem analysis. Information regarding the origin of the animals used in this study were unable to be obtained due to a privacy act of non-disclosure in part by the University of Guelph. Limbs were obtained for this study only after the owner or affiliation had given signed consent to donate the carcass.

### FE Model Development

#### Image acquisition and segmentation

Two right MCP joints (one healthy and one exhibiting signs of advanced OA–including bone sclerosis, erosion of the articular cartilage and deep pitting in the palmar aspect of the subchondral bone on MC3 determined through gross examination and micro-imaging) were harvested post mortem from female Standardbred horses (ages 5 and 7) for use in this study. Grading of the limbs was determined using the criteria established previously [4]. The specimens were scanned using micro-computed tomography (μCT) (GE Locus Ultra, GE Healthcare, Milwaukee, WI) at 120kV, 20mA and 900 views, generating isotropic voxels with 0.154 mm spacing. The scans included MC3 and P1 of the MCP joint without the proximal sesamoids (PS) (Fig 1). Each limb was oriented in the scanner such that the MCP joint angle was between 165-175°, similar to that found at the moment the hoof makes contact with the ground *in vivo* [21,22,23]. Joint angles were determined using software to measure the joint angle from photographs taken at a standardized location. The images were imported into Amira 5.2.2 (Mercury Computing Systems, Chelmsford, MA) where 3-dimensional (3D) bone surfaces were segmented, smoothed and exported in stereolithography (STL) format for importing into the FE software. The articular cartilage from the distal end of MC3 was initially included by extruding the surface to form a layer of elements where the elastic modulus = 10 MPa, Poisson’s ratio = 0.49 and the density = 1.05 g/cm^3^. The elastic modulus is representative of the instantaneous, or, dynamic modulus and was the appropriate choice given the high-speed and short duration that is characteristic of impact loading. The event duration during impact loading is too short to allow the interstitial fluid to flow relative to the solid matrix and hydraulic stiffening of the cartilage occurs. Under these conditions it has been shown that cartilage behaves as a single-phase, incompressible, elastic solid [24,25,26,27] and that the instantaneous modulus can be 10–20 times greater than that of the more often cited equilibrium and aggregate moduli [25,26,28,29]. In general, aggregate moduli for articular cartilage are in the range of 0.3–1.5 MPa [25]. Therefore, an elastic modulus of 10 MPa would represent a characteristic value of the instantaneous modulus calculated by applying the experimentally observed 10–20 times instantaneous-to-equilibrium modulus ratio, to the range of values reported for the equilibrium modulus. A Poisson’s ratio of 0.49 was chosen to model the incompressibility of cartilage given that cartilage behaves as an elastic solid at high loading rates [24,25,26,27]; significant amounts of lateral distortion of the cartilage occur under impact loading [6]; and that little to no volume change occurs during impact [28]. The cartilage layer was later purposefully omitted from the FE models as preliminary testing of our impact FE model, which included a cartilage layer, did not have a significant effect on the contact pressures or the von Mises stress in MC3. A recent study by Harrison et al. [1] demonstrated that their FE model of the equine MCP joint was highly sensitive to the thickness of the articular cartilage when loaded under static conditions, however the addition of a cartilage layer to our impact model may have been less sensitive under impact loading due to the viscoelastic nature of cartilage and the associated time dependent response of cartilage, which has been shown to stiffen significantly under high loading rates [30]. Given that there was no significant changes to the MC3 bone stresses during our preliminary testing using parameters more relevant for impact and that the addition of a non-subject specific cartilage layer in terms of thickness and geometry may have introduced errors, we chose to omit the cartilage from our FE models as the primary focus of this study was to examine the subchondral bone stresses under impact loading.

#### Metacarpophalangeal Mesh Development

Each of the 3D surface models were imported into an automated mesh generating software program (NetGen 4.9.13, Linz, Austria) where 4-node linear tetrahedral meshes were created. The meshes were then imported into Abaqus Explicit (v6.12, Dassault Systemes, Vélizy-Villacoublay, France) where the elements were converted into modified 10-node quadratic tetrahedral elements. A convergence analysis (described below) was performed to optimize mesh density against the number of elements (which increase inversely to density, with a concomitant increase in computing time).

#### Material Properties

**Fig 4:**
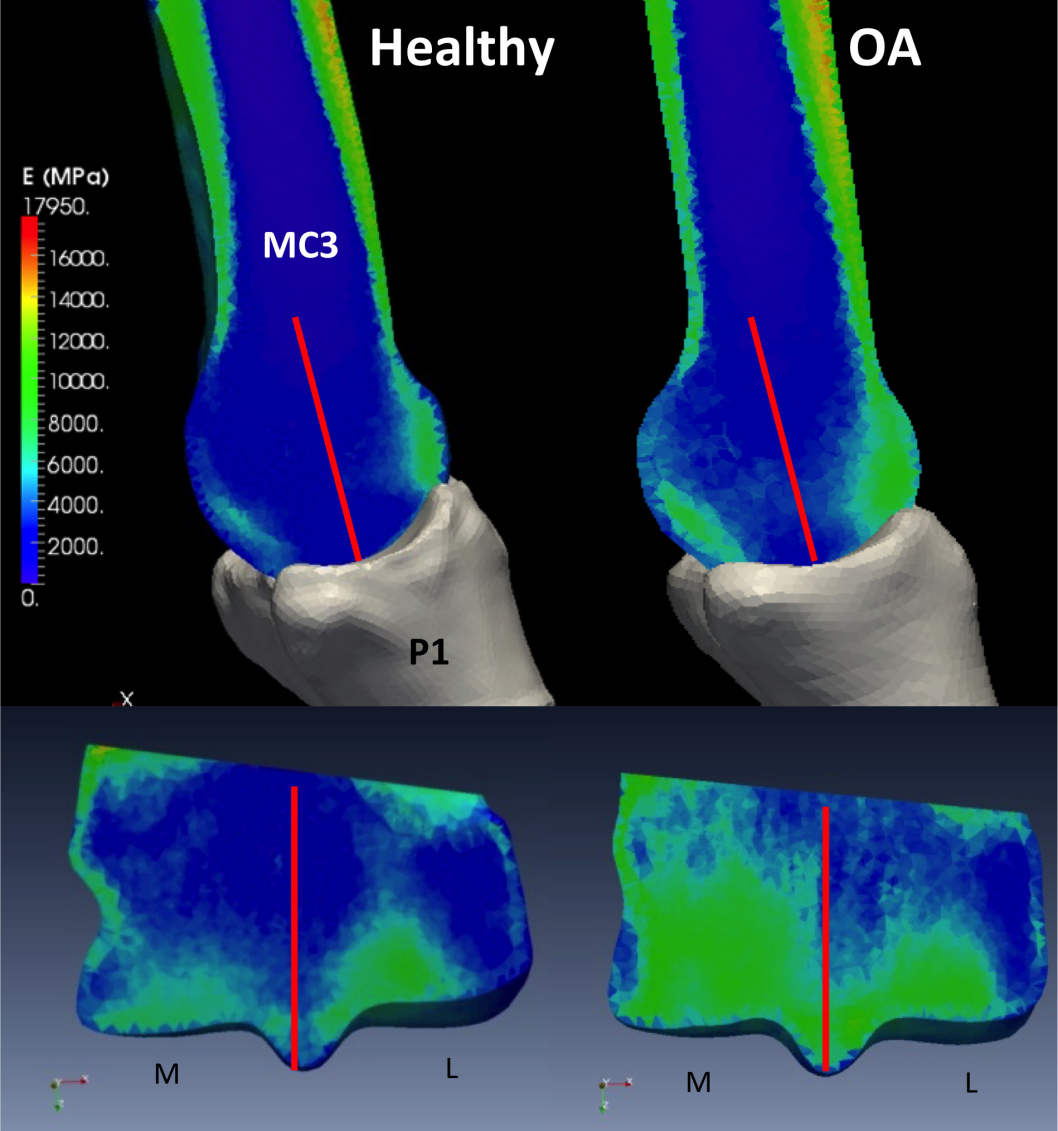
Mapped material stiffness on MC3 from μ-CT images. Upper images, parasagittal slices at locations indicated by a line on corresponding lower image. Lower images, frontal slices at locations indicated by lines on corresponding upper images. Bone stiffness represented by colour mapping on the images and corresponds to the colour scale which is measured in MPa.

The bone material properties (cancellous and cortical) were assigned on an element-by-element basis to the finite element models by mapping the bone density data that was obtained from the μCT images, using custom written software previously shown to provide accurate material property predictions [31,32]. The conversion was based on the density-modulus relation for equine metacarpus developed by Les et al. [33]:

$$E=9040\rho^{2.35}$$ (1)

where E is the elastic modulus (MPa) and ρ is the apparent bone density(g/cm^3^ or g/ml). Eq 1 was compared to the results of previously developed density-modulus equations for human bone (maximum apparent density approximately 2.0 g/cm^3^), which were extrapolated to the maximum apparent density of 2.47 g/cm^3^for equine specimens. Conversion of the image density to modulus was performed by: 1) converting HU for each voxel into equivalent ash density, 2) transforming ash density to apparent density, 3) applying the density modulus Eq (*1*), and 4) averaging the modulus values within each element in the mesh. These conversions provided the necessary material properties required to create a subject specific finite element model (Fig 4).

A set of dense elements (density = 500g/cm^3^, Young’s modulus = 16 GPa, Poisson’s ratio = 0.3) were specified at the proximal cut end of MC3 in order to increase the overall mass of MC3 to 5kg. This increased the mass of the model to approximate the effective mass of the distal limb that is associated with impact loading *in vivo* [34].

#### Impact Loading and Boundary Conditions

All impact simulations were performed using Abaqus Explicit software, by applying a velocity to all nodes of MC3, generating an impact with a stationary P1, the distal end of which was constrained in all directions. The velocity vector on each node (internal and external) had a magnitude of 3.55 m/s and was directed distally, parallel to the long axis of MC3. The time step size for the impact loading was defined at 0.25ms. These impact velocity parameters were based on data for a medium trot *in vivo* [34,35,36], and on the results of previous experimental *ex vivo* impact testing on the equine MCP joint [37].

General surface-to-surface contact was specified within Abaqus Explicit and the coefficient of friction between P1 and MC3 was set at 0.007 [38]. A linear interaction property was used, with the surface stiffness properties defined at 12 MPa/mm to allow for sufficient contact and “settling” of the contact surfaces before they separated on rebound. The total impact duration was defined at 3ms based on live animal data calculated from accelerations at high-speed [39].

#### Midstance (Static) FEM—Loading and Boundary Conditions

A simplified version of each FEM (MC3 only) was loaded to represent midstance (static) loading for the purposes of a direct comparison of stress magnitudes at impact loading in order to account for the subject specific material properties of the current FE models. The authors recognize that there are previous FE models of the MCP joint under midstance loading that include more detailed structures and have shown high contact stress in the palmar aspect of MC3 in the cartilage [1] and subchondral bone [2]. Previously existing models [1,2,19] and pressure data from previous *ex vivo* studies [2,40], that examined midstance loading in the MCP joint, were considered when establishing the loading magnitude for our simplified static FE models. While muscle forces were not modelled in the current FEM, MCP joint forces that occur during midstance as a result of muscle forces have been previously examined [1,2,40]. Based on the previously published data [2,40], we applied an average of the resulting loading forces directly to the surface of MC3. With the exception that P1 was not included in either of the midstance FE models all other aspects including element choice, mesh density and material properties were identical to the impact FE models. Simulations were performed using Abaqus Standard software and loading was applied using known static pressures on the surface of MC3 in the locations where P1 and PS make contact during midstance [40,41]. The pressures applied were chosen based on previous experimental *ex vivo* testing of joint pressures during midstance [2,40]. The proximal end of MC3 was constrained in all directions.

#### Convergence

Three models ranging from 33 thousand to 1.6 million second-order elements were created. All three models were identical in the element type, loading and boundary conditions and differed only in the mesh density and relative material property distribution. Stresses from the two coarser models were compared with those from the highest density mesh, using a convergence criterion of average stresses within ±5%.

### Testing to Determine FE Model Robustness

#### Sensitivity Testing

It has been shown that both bone stiffness and bone strength increase with strain rate [42]. When simulating impact loading, this factor requires consideration, as rate dependant loading could potentially have an effect on the results of the FE analysis due to the increase in material stiffness. Sensitivity testing was performed on the impact FE model to determine the effect of bone stiffening that occurs under an increased loading rate. The modulus values computed by software used to determine specimen-specific material properties (Fig 3) was multiplied by a factor of 1.5, based on the relationship established between strain rate and Young’s modulus in trabecular bone [43]. The effect of an increase in overall bone stiffness was determined by comparing the results of this model to the original impact FEM.

#### Experimental Testing

**Fig 5:**
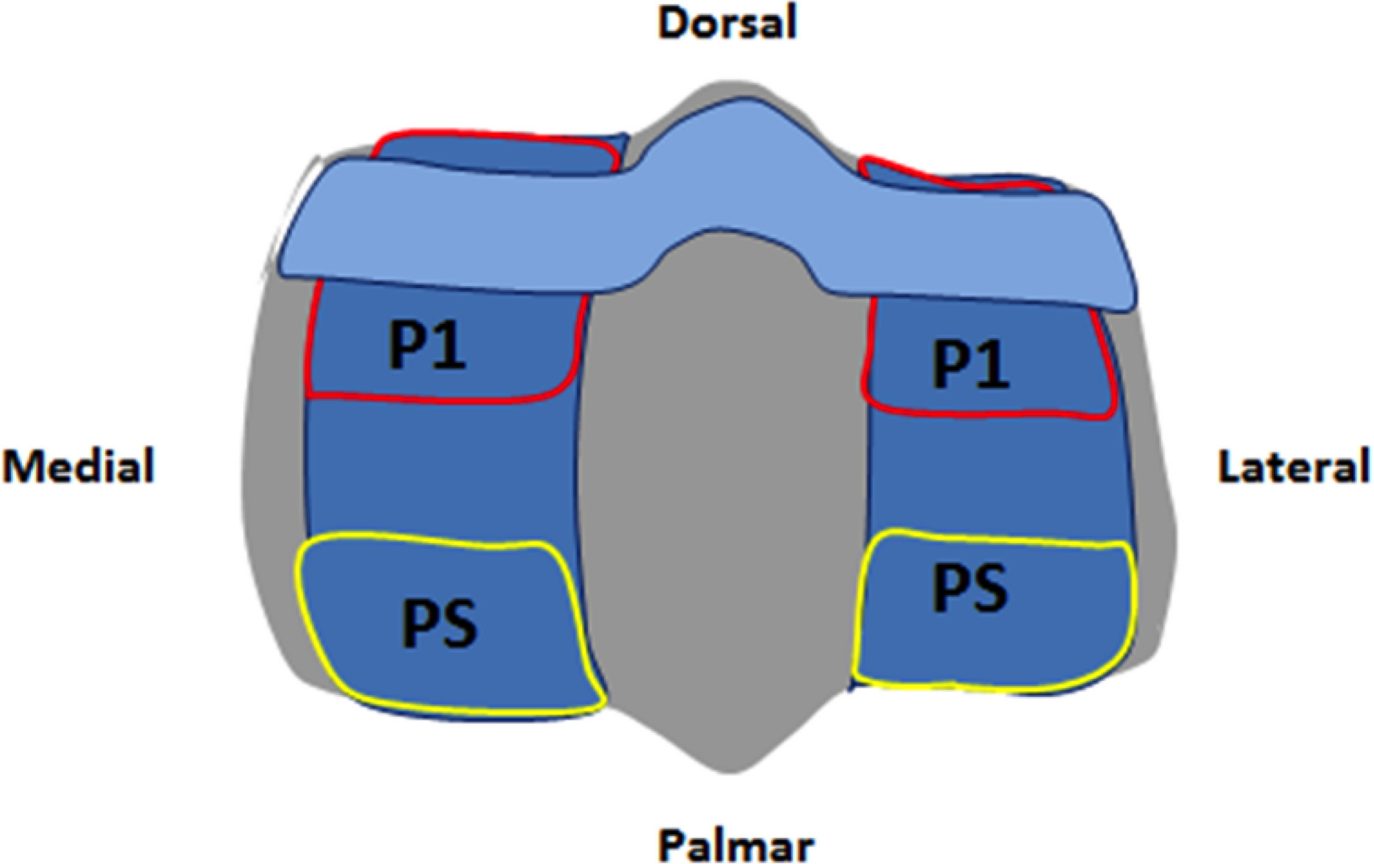
Placement of pressure film within the MCP joint during *ex vivo* testing. Diagram of the distal aspect of a third metacarpal. Blue areas indicate placement of pressure sensitive film within the MCP joint including P1 and PS contact area. Light blue area represents lateromedial film used to capture loading across the sagittal ridge.

Previous experimental *ex vivo* testing established a baseline of contact area and contact pressure in the MCP joint under impact loading [44]. A detailed description of the experimental apparatus and protocol can be found in [44]. Briefly, pressure sensitive film was placed within the MCP joint of equine cadaver forelimbs. Separate films were inserted over the lateral and medial condyles and transversely over the sagittal ridge (Fig 5). Subsequently, contact pressure and contact area between P1 and MC3 were estimated by the film under simulated impact loading from a 24 kg pendulum impact hammer. The height at which the hammer was released produced a repeatable impact velocity of 3.55 m/s at contact, which is within the normal *in vivo* range: 1.43 m/s for a medium trot to 7.2 m/s for a racing trot [36,38]. Forces and loading conditions that occur at a trot was chosen for simulation purposes in the current study as this is the typical racing and training gait for Standardbred racehorses.

#### Comparison between the impact FEM and experimental contact pressures and areas

**Fig 6:**
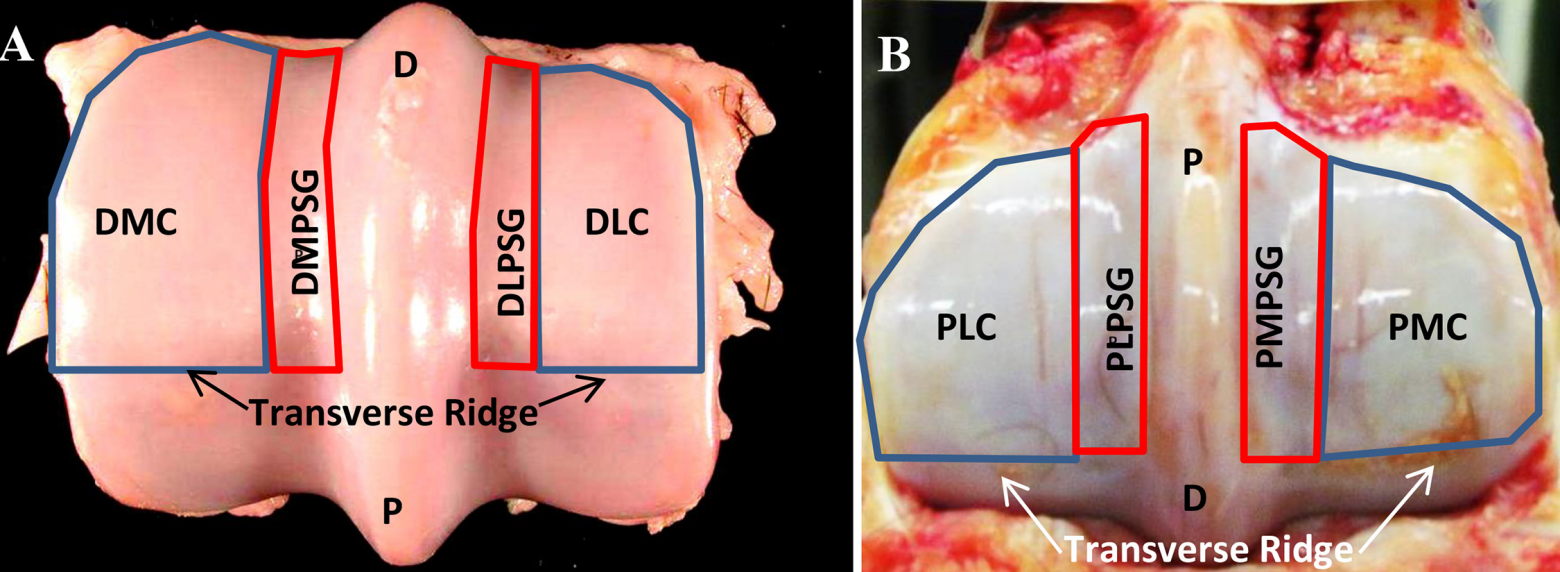
Regions of the distal end of MC3 used for the data analysis. A) Regions dorsal to the transverse ridge associated with contact made by first phalanx (P1). (image credit and permission for reprint, Easton 2012) as the parasagittal groove (PSG) and medial (M) and lateral (L) condyles Areas included all data. B) Regionspalmar to the transverse ridge, associated with contact made by the proximal sesamoids (PS). as the parasagittal groove (PSG) and medial and lateral condyles (PS). Areas included all data. D: Dorsal; DLC, DMC: dorsolateral and dorsomedial condylar regions; DLPSG, DMPSG: dorsolateral and dorsomedial regions of the parasagittal groove; P: Palmar; PLC, PMC: palmar lateral and palmar medial condylar regions; PLPSG, PMPSG: palmar lateral and palmar medial regions of the parasagittal groove.

The distal end of MC3 was subdivided into eight anatomical regions: dorsal lateral condyle (DLC), dorsal lateral parasagittal groove (DLPSG), dorsal medial condyle (DMC), dorsal medial parasagittal groove (DMPSG), palmar lateral condyle (PLC), palmar lateral parasagittal groove (PLPSG), palmar medial condyle (PMC), palmar medial parasagittal groove (PMPSG) (Fig 6). Images from the impact FE analysis were collected for the distal end of MC3 after contact was made between MC3 and P1 in all tests that were performed. A uniform sampling grid of 160 equidistant points was superimposed over the distal end of MC3 and scaled to accommodate differences in size and shape of the condyles. The calculated pressure at each sampling point was recorded, and the results were then grouped according to the anatomical regions in Fig 6, and averaged to provide location-specific contact pressures. Comparison of percent differences in contact pressures and areas were made between the results of the impact FE analysis and *ex vivo* experimental data collected previously.

### FE Data Analysis

Two mediolateral slices were extracted from a standardized location (5 mm dorsal and palmar to the transverse ridge on each specimen) on the on the distal end of MC3 in each model. The sampling grid of 160 equidistant points was superimposed over each lateromedial slice (scaled to accommodate differences in size and shape) and the von Mises stress was recorded at each point. Peak stresses were recorded and averages were calculated by anatomical location (palmar and dorsal), with reference to projections of the externally defined regions (Fig 6) into each section. Within-region maximal peak and average stresses were compared between healthy and OA bones in both midstance and impact loading.

## Results

### FE Model Robustness

#### Convergence

The results of the convergence analysis, using the von Mises stresses, indicated that the moderate resolution model obtained convergence within ±5% based on the average stress within a given location from a slice on the dorsal aspect of MC3 (Table 1). Based on these results, the moderate mesh was determined to produce a model with sufficient resolution of the material properties based on the micro-architecture (Fig 4) and the von Mises stress distributions that was within the convergence criterion (Table 1).

**Table 1:** Difference in average von Mises stress by location between the selected model (moderate) and the fine and course resolution models.

Resolution	Medial Condyle	Medial PSG	SR	Lateral PSG	Lateral Condyle
Coarse–Fine Fine	0.207	0.37	0.129	-0.034	0.153
Moderate–Fine Fine	0.050	0.036	-0.024	-0.043	0.041

PSG: Parasagittal Groove, SR: Sagittal Ridge

#### Sensitivity Testing

On increasing bone stiffness values by 50% in the impact models, average stress within each region of the distal MC3 condyle and parasagittal groove was between 1.0%– 1.09% greater (Table 2). Of the 160 points sampled, 12 (9.4%) had either an increase or decrease in stress among the increased bone stiffness model, compared to the original model, with the greatest percentage increase of 43% at an individual sample.

**Table 2:** Change in Average Dorsal Stress under Increased Bone Stiffness.

Density-Modulus Algorithm	Medial Condyle (MPa)	Medial PSG (MPa)	SR (MPa)	Lateral PSG (MPa)	Lateral Condyle (MPa)
E = 9040ρ^2.35^	12.80	8.47	6.11	7.55	10.87
E = 1.5(9040ρ^2.35^)	12.94	9.30	6.27	7.71	11.34
Percent Change	1.00%	1.09%	1.03%	1.02%	1.04%

Average stress by location for a standardized dorsal slice of the model impact with the original modulus-density algorithm and the modulus-density algorithm that accounts for an increase in bone stiffness that occurs under impact loading. Modulus equation from Les et al. 1994.

#### Comparison between Impact FEM and experimental contact pressures and areas

**Fig 7:**
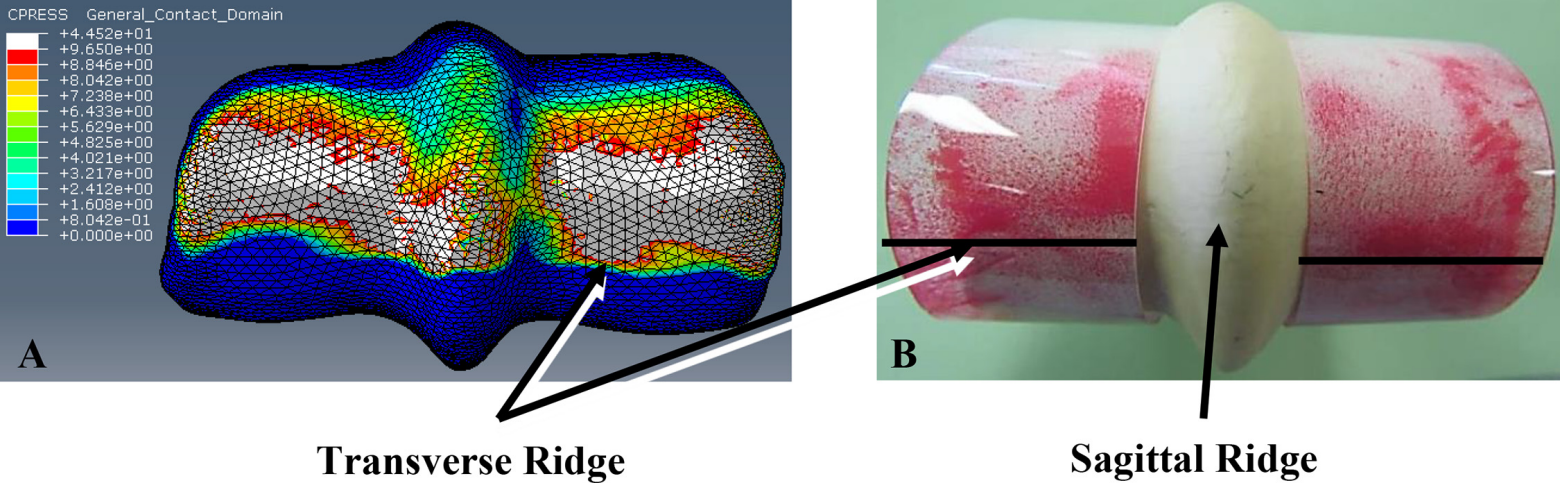
Comparison of FEM and experimental pressures. Distal end of MC3 of A) the FEM and B) the experimental pressure film indicating contact area and pressure (MPa) under impact loading at 3.55m/s (specimen in picture is for illustration purposes only and was not used in this study). B) The greater the intensity of the red staining on the experimental pressure film indicates a higher contact pressure at that location. Grey shading on FEM indicates an area that is over-limit based on magnitude measurable range of the pressure film.

**Fig 8:**
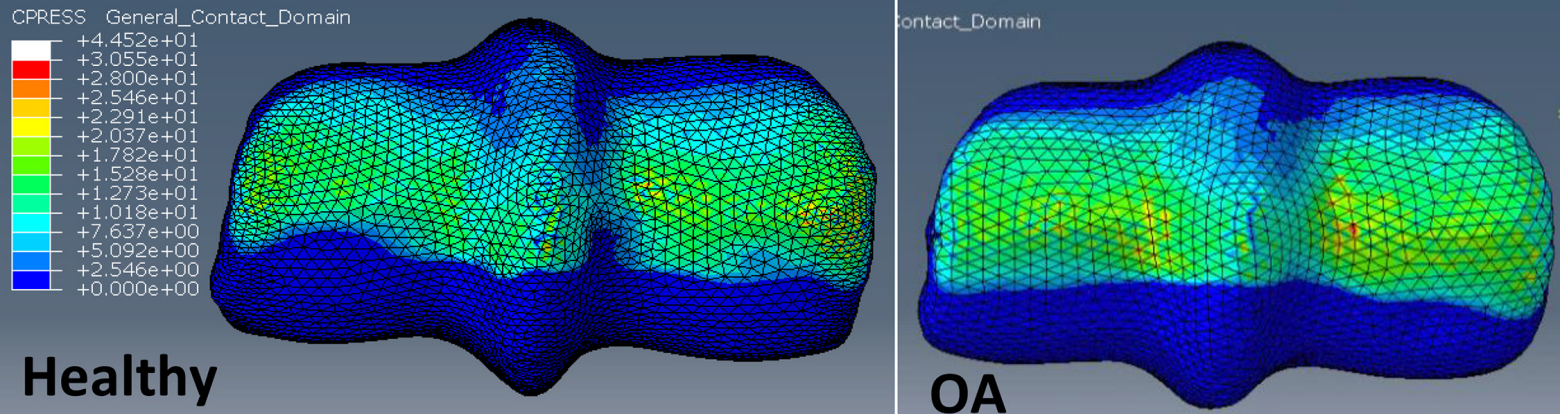
Comparison of OA and healthy FEM contact pressure. Distal end of the third metacarpal on the healthy FEM and OA FEM indicating contact area and pressure (MPa) under impact loading at 3.55m/s.

Contact area in the FE models was similar to the results found in the experimental *ex vivo* testing [36] with both exhibiting well-defined borders of contact on the dorsal aspect of MC3 from P1 up to the sagittal and transverse ridges and including contact across the sagittal ridge (Fig 7). Contact area in both the impact FEM and the experimental study indicated that loading occurred primarily in the DLC, DLPSG, DMC and DMPSG regions of the contacting surface of MC3. Average contact pressures from both the experimental pressure films and the impact FEM were found to be similar regardless of the region (Table 3), with the FEM results being consistently higher than the experimental by factors ranging from 34.8% to 52.3%. The highest average contact pressure occurred in the DMC of the healthy impact FEM (11.47 MPa), and in the DLPSG of the OA FEM (11.2 MPa) (Fig 8) and experimental film (7.26MPa). Average contact pressures in all other regions were within close range of each other by location in the FEM (range: 10.28–11.2MPa), and the experimental testing (range: 5.09–6.20 MPa). Maximum contact pressures (Table 4) occurred in the DMC for both FE models and the experimental testing with the highest pressure occurring in the healthy FEM (44.52 MPa).

**Table 3:** Average Contact Pressure by Location of Experimental and Impact FEMs.

	Medial condyle (MPa)	Medial PSR (MPa)	Lateral condyle (MPa)	Lateral PSR (MPa)
OA	10.66	10.69	10.28	11.20
Healthy	11.47	10.69	10.29	11.20
Experimental	6.61	5.09	6.20	7.26

Average contact pressure by region based on experimental *ex vivo* testing and finite element analysis of the healthy and OA impact models.

**Table 4:** Maximum Contact Pressure by Location of Experimental and Impact FEMs.

	Medial condyle (MPa)	Medial PSG (MPa)	Lateral condyle (MPa)	Lateral PSG (MPa)
OA	33.01	25.46	20.37	17.82
Healthy	44.52	25.46	20.37	17.82
Experimental	9.65	7.3	9.65	9.65

Maximum contact pressure by region based on experimental *ex vivo* testing and finite element analysis of the healthy and OA impact models.

### FEM Results from Analysis

#### Impact FEM—Stress Distribution

**Fig 9:**
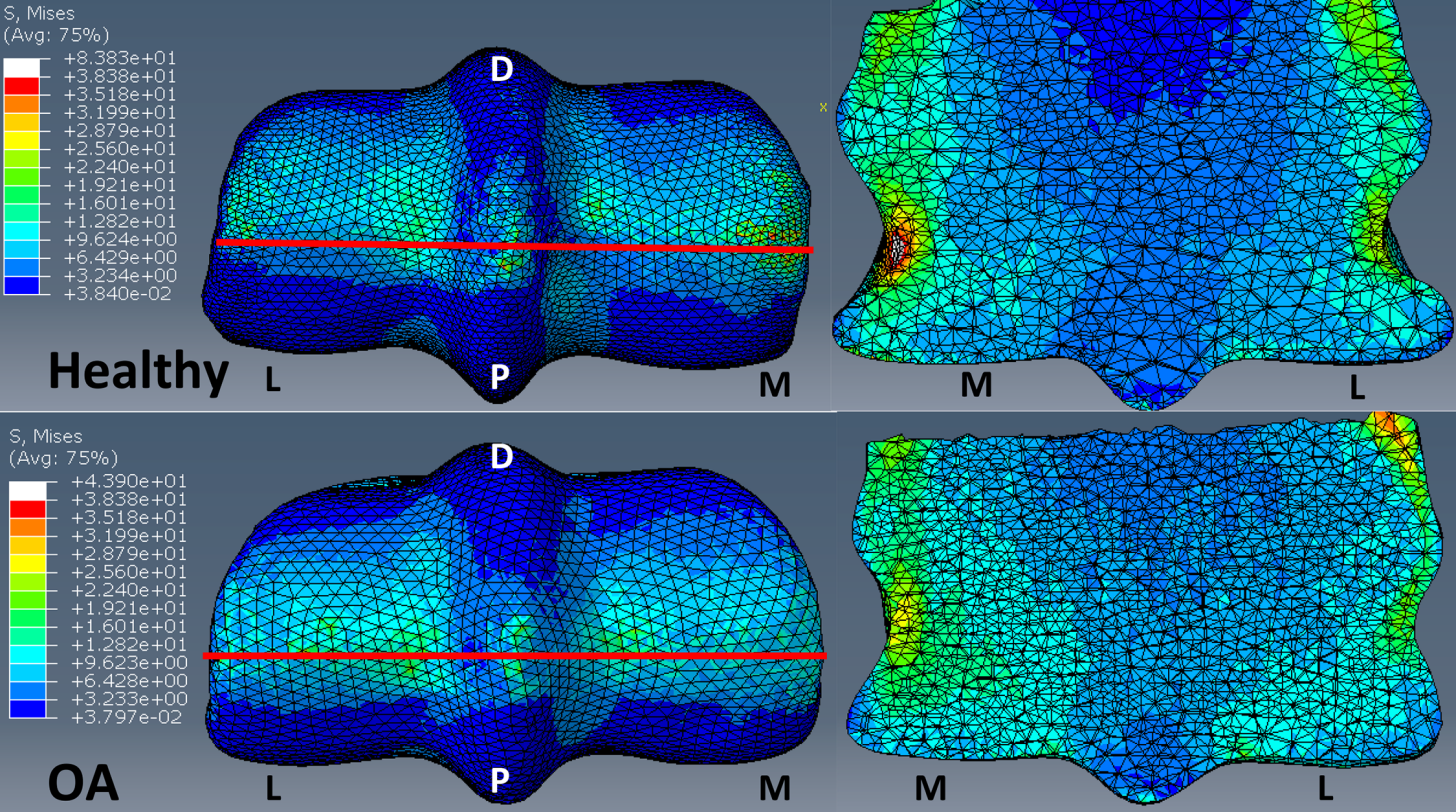
Comparison of OA and Healthy FEM surface stress. Comparison of von Mises (MPa) surface stress (left) and a lateromedial slice (red line indicates area where slice was taken) of von Mises stress (right) between healthy and OA models under impact loading. M: Medial, L: Lateral

**Fig 10:**
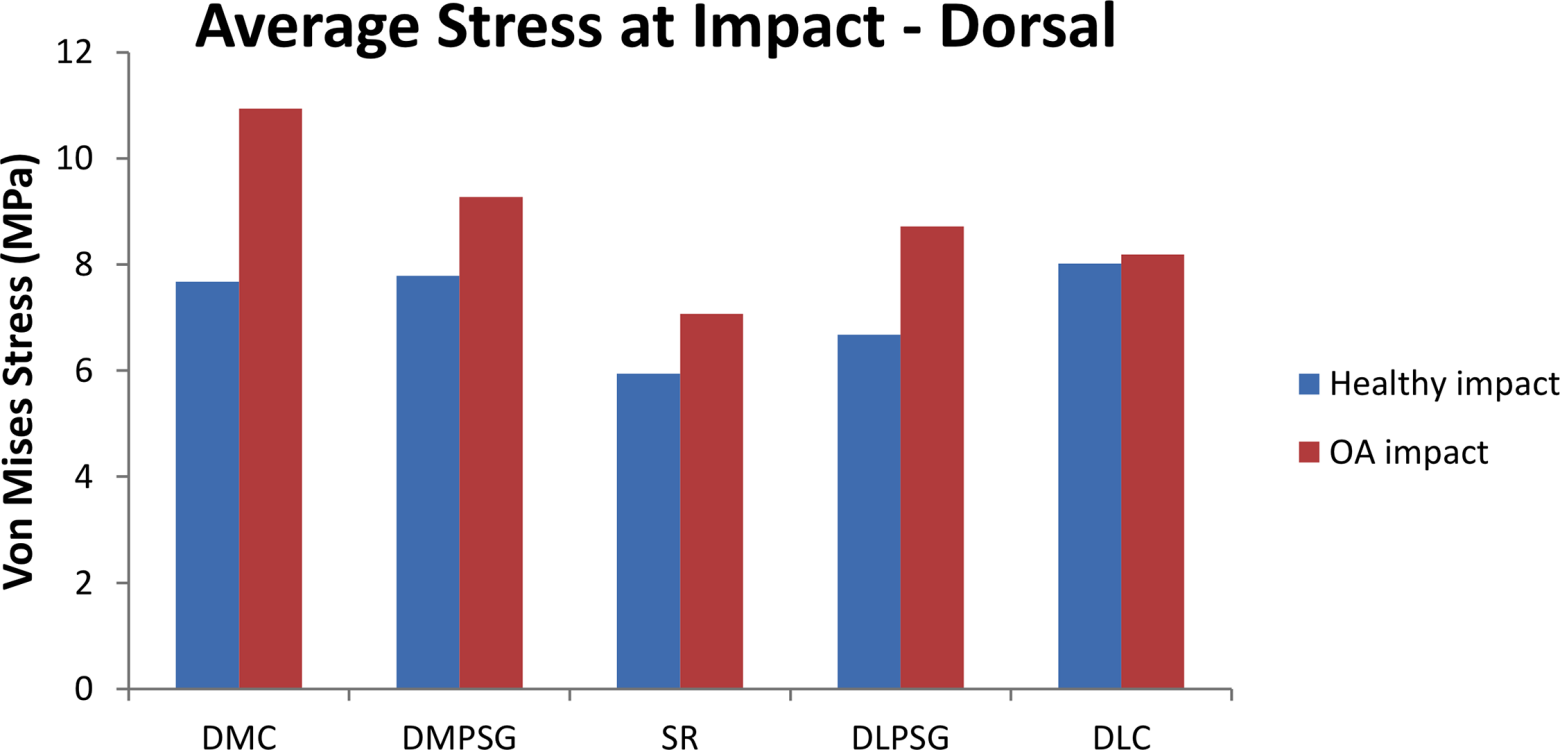
Comparison of the average von Mises stress (MPa) at multiple locations on a lateromedial slice in the dorsal region of MC3 between the healthy and OA impact models. DMC: Dorsal Medial Condyle, DMPSG: Dorsal Medial Parasagittal Groove, SR: Sagittal Ridge, DLPSG: Dorsal Lateral Parasagittal Groove, DLC: Dorsal Lateral Condyle

Surface von Mises stress by region were similar in distribution between the healthy and OA models, however, the overall stresses were greater in the OA model (Fig 9, left panels). The average von Mises stresses across all regions, in both locations (palmar and dorsal) was greater in the OA model when compared to the healthy model (Fig 9 –right panels and Fig 10). The maximum von Mises stresses were located in the DMC in the healthy (12.8 MPa) and OA (14.1MPa) models, and were lowest in the PMPSG and PLPSG for the healthy (5.94 MPa) and OA (7.07 MPa) models, respectively.

#### Midstance (Static) FEM–Stress Distribution

**Fig 11:**
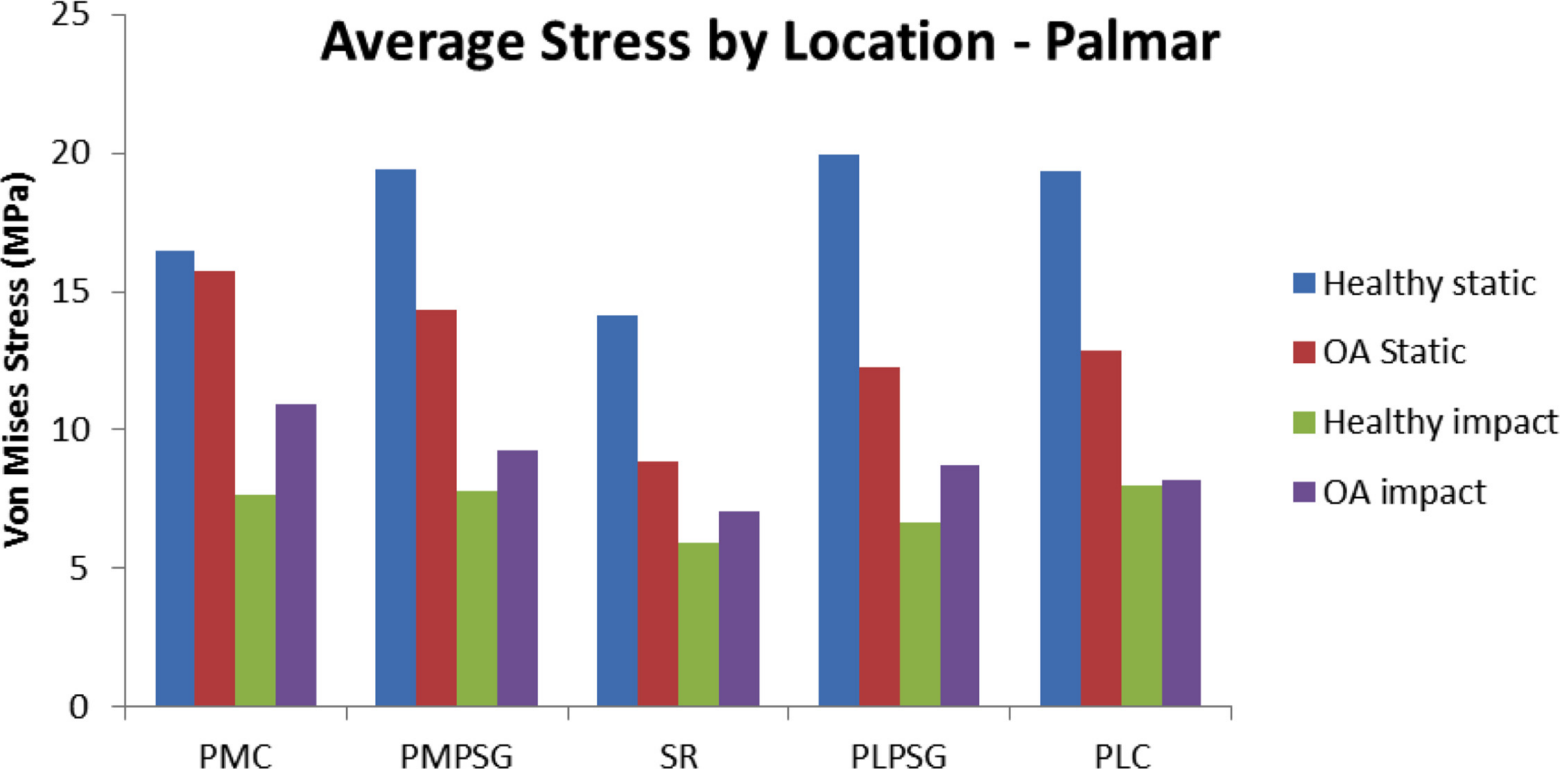
Comparison of average von Mises stress (MPa) on a lateromedial slice in the palmar region of the third metacarpal across location between impact and static loading in the healthy and OA models. PMC: Palmar Medial Condyle, PMPSG: Palmar Medial Parasagittal Groove, SR: Sagittal Ridge, PLPSG: Palmar Lateral Parasagittal Groove, PLC: Palmar Lateral Condyle.

The highest peak von Mises stress was 38.38 MPa in the PLC, while the highest average von Mises stresses was 19.38 MPa in the PLPSG and 19.94 MPa in the PMPSG of the healthy midstance FEM (Fig 11). The healthy FEM had higher peak von Mises stresses (Table 5) in both locations and across all regions (range 0%– 49.7% greater) compared to the OA midstance FEM. The healthy midstance FEM had higher average von Mises stress (Table 6) in both locations and across all regions (range 0%–38.6% greater) compared to the OA midstance model with the exception of the DLPSG (7% less than the OA midstance FEM).

**Table 5:** Maximum Stress for Static FE Models.

	Medial condyle (MPa)	Medial PSG (MPa)	SR (MPa)	Lateral PSG (MPa)	Lateral condyle (MPa)
**PALMAR**					
Healthy	35.20	32.00	22.44	28.82	38.38
OA	25.67	19.31	12.95	19.31	19.31
**DORSAL**					
Healthy	22.44	16.06	9.69	12.88	32.00
OA	22.44	9.78	9.78	9.78	25.67

Maximum von Mises stress by location and region for midstance (static) FE models.

**Table 6:** Average Stress for Static FE Models.

	Medial condyle (MPa)	Medial PSG (MPa)	SR (MPa)	Lateral PSG (MPa)	Lateral condyle (MPa)
**PALMAR**					
Healthy	16.48	19.38	14.15	19.94	19.34
OA	15.71	14.34	8.89	12.24	12.85
**DORSAL**					
Healthy	13.41	10.57	7.65	8.38	14.54
OA	12.65	9.38	7.65	8.98	12.04

Average von Mises stress by location and region for midstance (static) FE models.

## Discussion

While there have been several previous studies examining stresses during midstance loading using FE models [1,2,16,17,18,19,20], this is the first study to examine impact loading within the equine MCP joint using finite element analysis. In the current investigation, specimen-specific FE models were developed to examine the stresses that occur in the MCP joint under primary impact at a medium trot in order to compare the stresses within the distal end of MC3 in a healthy and OA model under 1° impact and midstance loading. Overall, the results indicated that loading that occurs during 1° impact produces similar stresses within the subchondral bone on the dorsal aspect of MC3 when compared to midstance loading. Based on the two FE models that were derived from specimens which exhibited normal bone density and focal increases in bone density associated with OA, the results indicate that the change in bone density had an effect on the resulting stress magnitude and distribution when loaded under 1° impact and midstance conditions.

### Comparison between impact FE models and experimental data

Predictions of contact areas and regions of high contact pressures from the FE models were in strong concordance with experimental results [44]; The FE models predicted higher contact pressures than the experimental data, but this difference is likely due to the inability to accurately measure maximum contact pressures in the experimental testing (see limitations for further detail).

### Comparison between Current Midstance FE Model and Other FE Models

Although loading for the current FE model for midstance at a trot was determined using the results of previous *ex vivo* testing and FE models [2,40], to the best of the authors knowledge, there are no FE models of the equine MCP that report bone stress in the MC3 under trotting loads. The study by Brama et al. 2001 [40] did not involve the active muscle forces and was therefore used for comparison to the current FE static models as it closely replicated the conditions that were modelled in the current study. In the study by Harrison et al. 2014 [1], the authors reported average and maximum cartilage contact pressure and von Mises stress under trotting loads in the equine MCP joint. This model was highly detailed and included many structural components that were loaded using representative muscular forces, however the reported mean pressures on MC3 from P1 (41–54 MPa) were almost two times greater than the *ex vivo* joint pressures measured by Brama et al. [40] (approximately 19 to 20 MPa), possibly due to the addition of the active muscular forces. Given that our static model was loaded using an average from the results of previous *ex vivo* testing (which did not include the active muscular forces) [2,40] and that the FE results from the study by Harrison et al, 2014 [1] reported *ex vivo* contact pressures almost two times greater than the previous *ex vivo* studies without active muscle forces, this study was not used for comparison to the current study.

### FE Analysis

#### Surface Contact Pressure and Contact Area under Impact Loading

The contact area associated with impact loading was found to occur in the dorsal aspect of MC3 compared to midstance where loading occurred in both the dorsal and palmar aspect due to contact with P1 and PS (Fig 7). The orientation of the distal limb at 1° impact allows for an MCP joint angle of approximately 165-175° based on *in vivo* kinematic data [21,23]. As was determined experimentally [44], the PS does not make significant contact with MC3 during this phase of the stance. There was no appreciable difference between contact pressure in the healthy and OA impact FE models across all regions. This was to be expected as the differences in bone density appeared to occur beneath the bone surface within the underlying subchondral bone of the specimens used in this study (Fig 4).

#### Stress Distribution—Healthy vs. OA under Impact Loading

In the current study, impact loading was found to produce higher stresses in the OA model when compared to the healthy model. Analysis of the MCP joint of racehorses with severe OA found that the subchondral bone plate was weakened (effectively decreasing the shock absorbing capabilities) and the trabecular bone was stronger (likely as a result of the increased load being transmitted to the trabecular bone) when compared to horses with mild OA [13]. Bone stiffness has been shown to be proportional to the apparent density such that an increase in bone density leads to an increase in bone stiffness [45]. The viscoelastic response of bone suggests that the stiffness is also related to the loading rate, in that an increased loading rate has been shown to increase the stiffness of trabecular bone [43]. Therefore, the higher stresses in the OA model under impact may be related to the increased bone density observed in the specimen with OA, leading to increased stiffening of the bone, particularly when loaded rapidly under impact loading conditions.

#### Stress Distribution—Impact Loading vs. Midstance Loading

**Fig 12:**
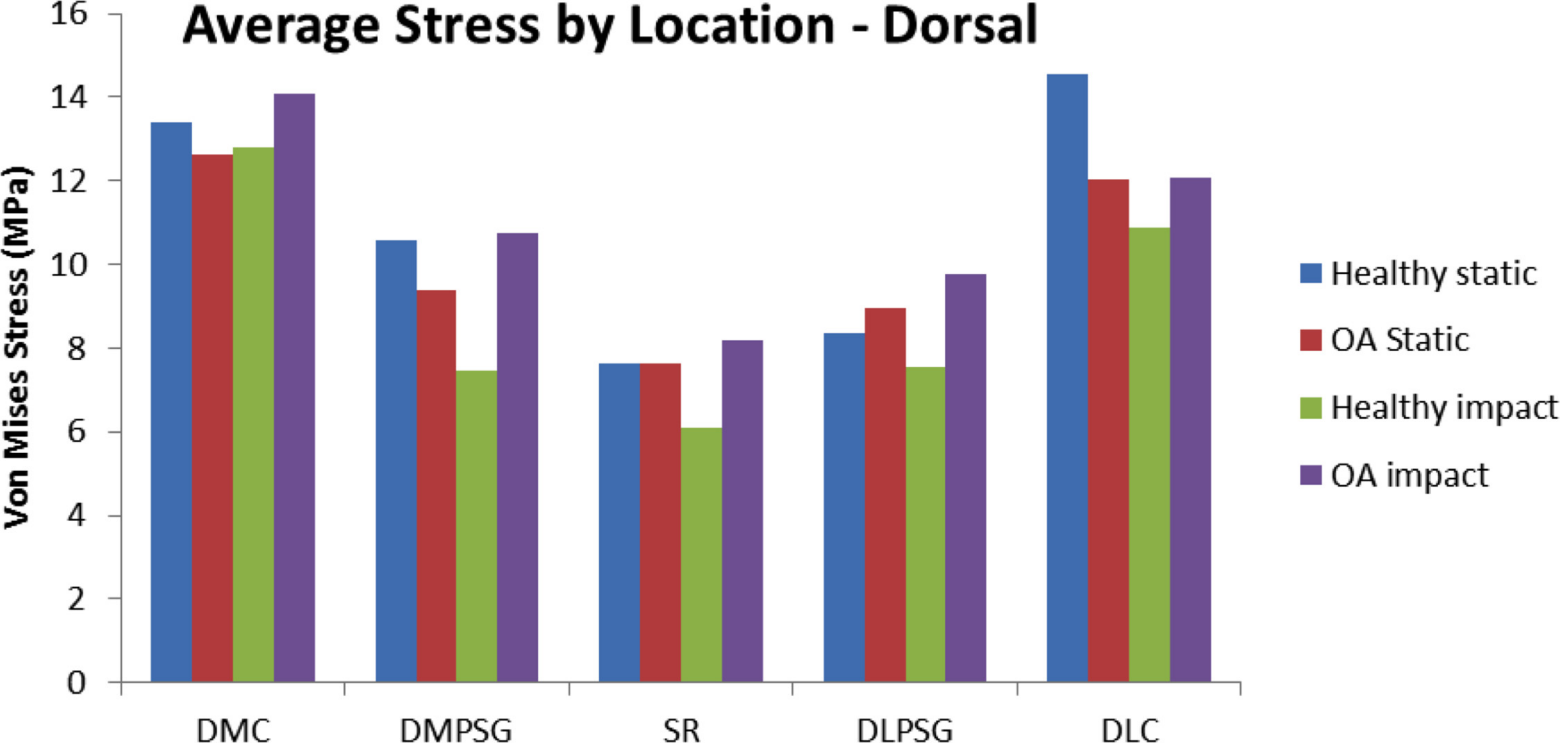
Comparison of average von Mises stress (MPa) on a lateromedial slice in the dorsal region of the third metacarpal across location between impact and static loading in the healthy and OA models. DMC: Dorsal Medial Condyle, DMPSG: Dorsal Medial Parasagittal Groove, SR: Sagittal Ridge, DLPSG: Dorsal Lateral Parasagittal Groove, DLC: Dorsal Lateral Condyle.

The highest von Mises stresses differed by location and region on MC3 when comparing impact and midstance loading (Figs 11 & 12). Stresses in the palmar aspect of MC3 was considerably greater under midstance loading with von Mises stresses that were approximately 21% -66% greater than in impact loading by region (Fig 11), due to the loading that occurs from the PS. In the dorsal aspect of MC3, the stress magnitudes were similar between impact and midstance loading (Fig 12) with the OA impact model showing greater von Mises stresses by region in a range of 0%–14% when compared to the OA midstance model. Although it was determined experimentally that the PS do not have a significant role in loading on the MC3 under impact loading [37], it has been determined that the flexor and extensor tendons produce opposing forces in an effort to stabilize the MCP joint prior to impact [46]. While there is no appreciable joint rotation [47], it is possible that surface contact between MC3 and PS at the moment of impact may occur. This was not accounted for in our model and should be considered for future impact FE models.

Impact loading in the distal equine limb has been shown to produce high-magnitude, high-frequency signals that contain significant energy extending up into the mid-diaphysis of MC3 [37]. By comparison, the high forces generated during midstance loading (from both the action of P1 and PS on the distal end of MC3) is a combination of compressive and shear loading that creates high stresses within a localized area [1,2]. While our results indicate that the loading conditions that occur during primary impact can produce stress magnitudes that are similar to those found during midstance loading, the area in which these higher stresses occur under impact loading, are not typically found to be associated with areas of injury in mechanically induced OA.

### Biomechanical Loading and Bone Adaptation

Previous authors have suggested that the high stresses generated during midstance are responsible for the changes in bone density within the MCP joint of performance horses [47,48], however there is evidence to suggest that vibration frequency associated with impulse loading is the primary stimulus for bone adaptation [49,50]. Bone responds to mechanical stimuli by modeling and remodeling to strengthen the areas in the direction that the primary load is applied and repair damaged bone [2,51,52]. It has been shown that areas consistently in contact under higher loads during midstance loading are associated with increased subchondral bone density, suggesting that SCB remodels and adapts to the applied load [2,53]. The results from the current study indicate that impact loading produces stress magnitudes that are similar to those found under midstance loading. The areas of high stress loading under impact loading were not found occur in areas commonly associated with OA injury, and therefore are unlikely to be implicated in the role of OA within horses. While it was not measured in the current study, it is possible that the transition from high-acceleration impact loading to high-magnitude midstance loading may be involved in the induction of OA injury. This transition phase (known as secondary impact) is associated with rapidly increasing forces and decreasing accelerations [54]. Both high magnitude loading and high acceleration loading have been shown to associated with changes in bone tissue [47,48,49]. Therefore, given that secondary impact has been shown to have both loading conditions and is a transition phase from one extreme type of loading (high-acceleration, low magnitude) to midstance loading (low or no accelerations, high magnitude) and has not been well studied, it is the final phase of the stance which should be considered for future investigation in the context of injury.

### Limitations

Despite our best efforts to capture the maximum joint pressures in the MCP joint under impact loading, our results found that the pressures exceeded the range limit of the pressure film (2.5–10 MPa). An attempt was made to measure the maximum MCP pressures under impact loading using the next available film with a range of 10–50 MPa, however preliminary testing found that this range of film was unable to capture and differentiate the pressures under the simulated impact loading conditions, likely to due to film insensitivity associated with the greater range.

Although we used data from previous *ex vivo* testing when loading the static model in the current study, we did not perform any static mechanical testing of our own. Therefore, it is possible that the results from our static FE model may have been affected as they were loaded using the average of previously existing *ex vivo* data that were not specific to the specimens used in the current study.

Not all structures located in the MCP joint of the live animal were included in the FE models. Previous *ex vivo* data has shown that the PS does not make significant contact with the distal end of MC3 during impact [44]. It has also been suggested that the use of spring elements to model tendons and ligaments tend to oversimplify the model and introduce error by neglecting to account for tendon or ligament structure and loading rate. Although it has been shown that the flexor and extensor tendons in the equine distal limb maintain some tension to properly align the joint at impact, these forces are minimal and there is no joint rotation at this moment [46], so the net tendon forces were assumed to be zero. The inclusion of the flexor and extensor tendon forces of equal and opposite moment around the MCP joint would have increased the forces applied to the joint surface. By omitting these forces in our models, the results provide insight into the forces occurring solely as a result of impact. The cartilage was omitted as preliminary testing of the impact FE model that included a cartilage layer did not have a significant effect on the contact pressures or the von Mises stress in MC3. The midstance FE model did not include the cartilage, as it has been shown that static loading of the MCP joint is highly sensitive to the thickness of the articular cartilage [1], and therefore the inclusion may have introduced errors as we did not measure the subject specific cartilage thickness. However, additional testing is recommended to further examine the effect of subject specific articular cartilage in the MCP joint specifically under impact loading.

Due to the complexity in the materials and structures involved when modeling biological systems, some simplifications and input assumptions are necessary. There is a lack of existing evidence within the literature on the performance of structures such as the tendons and ligaments under impact loading and inclusion of these structures without adequate knowledge of the response under impact loading could introduce error into the model. Such detail is not entirely necessary when modeling the equine MCP joint at impact because the primary forces on MC3 are directed across the articular surfaces with minimal shear loading. With respect to the subchondral bone, although there are well noted changes to the bone density in horses with OA, there are likely changes to the bone anisotropy which may contribute to the increase in bone stiffness. We were unable to assess the effect of the changes in bone anisotropy associated with the OA specimen due to the resolution of the microCT imaging and the resulting FE model roughness. Although the change in bone anisotropy in the current study were unable to be addressed, this is consistent with the previous FE models of the equine MCP joint who were also unable to account for bone anisotropy. The effect of changes in bone anisotropy associated with OA under impact and midstance loading remains unanswered and requires further investigation.

Although care was taken to create a displacement boundary condition that was well away from our area of interest, some of the resulting high stresses found on the proximal end of MC3 were likely due to the constraint occurring from the boundary condition rather than the contact and loading stresses. These were away from the regions of interest at the distal articular surface and the model showed good agreement to the experimental results in terms of contact area, so this was likely not contributing to the contact pressures calculated at the contacting surfaces.

## Conclusion

There are many factors that play a role in biomechanical loading and joint injury, including: individual conformation, footing surface, neuromuscular fatigue and speed and duration of training and racing [14,54]. Impact loading has been shown to be associated with high accelerations and results from the current study suggest impact loading creates stresses comparable to those found in midstance loading on the dorsal aspect of MC3 under simulated trotting conditions. Although the stress magnitudes were found to be similar between impact and midstance loading, the areas of high stresses under impact loading were not located in sites commonly associated with OA injury. Although impact loading may not be involved with the initiation of OA or OA injury, secondary impact, the transition phase from impact to midstance loading should be considered for future study. Given that similar stress magnitudes are created under impact and midstance loading and that the conditions under which these stresses are created are on the extreme ends of the loading spectrum, future study of the rapid transition from impact loading to midstance loading may provide further insight into examining the potential for injury in the equine MCP joint.
